# Proactive and Reactive Aggression Subgroups in Typically Developing Children: The Role of Executive Functioning, Psychophysiology, and Psychopathy

**DOI:** 10.1007/s10578-017-0741-0

**Published:** 2017-07-05

**Authors:** Nicholas D. Thomson, Luna C. M. Centifanti

**Affiliations:** 1 Department of Psychology, University of Durham, South Road, Durham, Durham DH1 3LE UK; 2 Department of Clinical Psychology, Liverpool University, Brownlow Hill, Liverpool, L69 3GB UK

**Keywords:** Psychopathy, Respiratory sinus arrhythmia, Executive function, Proactive aggression, Reactive aggression

## Abstract

This study aimed to assess whether groups of aggressive children differed on psychopathic traits, and neuropsychological and neurobiological measures of prefrontal functioning consistent with the objectives of their aggression—reactive or proactive. Including 110 typically developing children (9–11 years), a latent class analysis identified a low aggression group, a high reactive aggression group, and a mixed (high reactive and proactive) aggression group. Results show high callous–unemotional traits and low resting respiratory sinus arrhythmia increased the likelihood of children being in the mixed aggression group, when compared to the reactive and low aggression groups. However, deficits in planning and inhibitory control increased the likelihood of children being in the reactive aggression group, when compared to the mixed and low aggression groups. Executive functioning deficits did not differentiate the mixed group from the low aggression group. These findings highlight psychobiological and executive functioning differences that may explain heterogeneity in childhood aggression.

## Introduction

Middle childhood is a period of dramatic social, biological, and cognitive change [1, 2]. Healthy social and emotional functioning comes with significant developmental changes. Although aggression is prevalent during early childhood, in typically developing children, these behaviors decline with age [3, 4]. However, a small number of children (5–10%) continue to show aggressive behavior that continues through the teen years into adulthood [5, 6]. Although research on aggression has largely focused on drawing etiological and phenomenological distinctions between different types of aggression, there has been a growing interest in identifying children who pose the greatest risk of aggressive behavior. In particular, it is not uncommon to find youth who only engage in high levels of aggression that is in response to perceived provocation or threat (reactive aggression [7]). Yet, youths who only engage in aggression to achieve a goal (proactive aggression [7]) are rare [8]. Some youths use both types of aggression [7, 9]. Cognitive abilities differentiate proactive aggression from reactive aggression, yet studies looking at the psychophysiological and neuropsychological functioning related to aggression have been sparse [2, 10]. The present study tested whether and how measures of prefrontal functioning: executive functioning, psychophysiology, and psychopathy differentiated groups of aggressive children.

### Reactive and Proactive Aggression

Proactive aggression is goal-directed and predatory [11], and has been linked to greater psychopathic traits and lower psychophysiological activity (e.g., low resting heart rate [12]). The neuroscience of proactive aggression has been suggested to be more complex than that of reactive aggression, because of the cognitive demands the behavior requires [13]. Proactive aggression requires planning and can be a drawn-out process [13]. In contrast, reactive aggression, characterized as a hostile response to minor or perceived provocation or threat, has been associated with poor behavioral control and emotional hyper-reactivity [14].

Although distinctions of aggression subtypes have clear empirical value, there have been several criticisms over the variable-centered nature of examining reactive and proactive aggression, and failing to account for the co-occurrence of reactive and proactive aggression [15]. A person-centered approach, used to identify groups of adolescents based on their use of proactive and reactive aggression, identify a low aggression group, a reactive aggression group, and a “mixed group” who show both reactive and proactive aggression [8]. The mixed aggression group typically makes up about 10% of community adolescents [16], which is consistent with the proportional estimates of those children who continue to be highly aggressive into adulthood [5, 17]. Community samples of adolescents who exhibit only reactive aggression are typically the largest of the aggressive groups identified (33% [9]). To show the validity of such groups, adolescents in the mixed aggression group have higher arrest rates, delinquency, and increased emotional and behavioral dysregulation when compared to nonaggressive and reactively aggressive youth [9].

A person-centered approach has yet to be conducted in younger children. Identifying groups of children based on their use of reactive and proactive aggression is essential for understanding individuals [18]. Examining group differences on neurobiological functioning, executive functioning, and personality may help explain the mechanisms by which children come to be reactive and proactive aggressors.

### Psychopathy

Children who use a mix of proactive and reactive aggression show high levels of psychopathic traits. In youths, psychopathy has been associated with proactive aggression but not reactive aggression [19]. However, differences emerge with the dimensions of psychopathy. Psychopathic traits in youth consists of three dimensions: callous–unemotional (e.g., callous lack of empathy, lack of guilt/remorse, unemotional), narcissism (e.g., egocentric, superficial, and charming), and impulsivity (e.g., risky and dangerous behavior, blames others for mistakes, and proneness to boredom [20]). The impulsivity dimension has been found to relate to reactive aggression [21], and the narcissism dimension relates to both proactive and reactive aggression [22]. However, the callous–unemotional dimension has been shown to designate a particularly aggressive subgroup—displaying high levels of both reactive and proactive aggression, and more likely to develop severe antisocial behavior into adulthood [23]. Thus, children classified in a mixed versus reactive subgroup would be expected to differ on the dimensions of psychopathic traits just like older youths do.

Youth with conduct problems and callous–unemotional (CU) traits show reduced grey matter volume in the left orbitofrontal cortex (OFC), whereas youth with low CU traits and conduct problems do not display these deficits [24]. The OFC is part of the paralimbic region, which is involved with autonomic and response inhibition functions, and is shown to have an important role in social and emotional behavior, and risk-taking [25]. Thus, youths with a combination of CU traits and behavior problems, like aggressiveness, may be neurobiologically distinctive from youths with behavior problems but without CU traits [26]. Further, CU traits have been associated with low psychophysiological reactivity [27], poor recognition of fear in others [28], and a callous motivation for hurting people [29]. Of note, then, children with CU traits may display particular emotional poverty that is not characteristic of the other dimensions of psychopathy. Since the mixed aggressive group are expected to be higher on CU traits, they may also show low psychophysiological functioning, which can be considered a marker of prefrontal cortical functioning [1].

### Psychophysiology and Aggression

Respiratory sinus arrhythmia (RSA) is used to index parasympathetic influence on heart-rate variability via the vagus nerve [30]. High resting RSA represents greater vagal control of the heart, which enables individuals to adapt in the face of a challenge [30–32]. Conversely, low resting RSA indicates reduced myelinated vagal control that may interfere with the ability to regulate behavioral and emotional states [30]. Resting (often called “baseline”) RSA is a correlate of physiological and behavioral adaptation [33, 34]. Although RSA reactivity reflects physiological change because of situational contexts [30, 35], resting states mark a child’s biological disposition to respond to the environment, internally and externally, prior to the occurrence of an event such as attention, emotion regulation, and social communication [30, 36]. Beauchaine [1] proposed RSA as an efferent marker for PFC functioning, and thus plays an integral role in social development. Indeed, prior research has shown resting RSA to be lower among children or adolescents with externalizing problems [37, 38], and adolescents high on CU traits [39]. Therefore, resting RSA may serve as neurobiological marker to differentiate groups of aggressive children.

### Executive Function (EF) and Aggression

One proposal is EF operates hierarchically, concurrently, and interactively to influence goal-directed behaviors [40, 41]; thus, intact EF may facilitate the goal-directed aggression associated with proactive aggression. Indeed, EF is integral to a child’s social development. Deficits in EF have a fairly robust association with aggression and peer problems [4, 40, 42]. Specifically, during preadolescence, poor planning and inhibitory control has been associated with reactive aggression during this developmental period (9–12 years [40]). We argue, when compared to reactively aggressive children, children who are proactively aggressive should be better able to plan and inhibit their behaviors, and manage to cognitively switch between operations in order to achieve goal-directed action. EF skills are useful in keeping attention over time (working memory), selective attention (inhibitory response), and attention to switching between tasks, operations or mental states (cognitive flexibility [2, 43, 44]). Neuroimaging and lesion studies have indicated that PFC functioning is integral to EF skills. In particular, performance on planning [45], cognitive flexibility [46, 47], concept formation [48], and inhibitory control [49, 50] have been linked to the dorsolateral prefrontal cortex (dlPFC), an area of the brain found to be associated with regulation of aggressive social behavior [51].

Different abilities in EF may explain why some are able to adeptly respond to social situations for personal gain (e.g., manipulation), and why others fail to inhibit aggressive behavior in response to perceived provocation. In particular, it may be that children who display only reactive aggression have more global executive function difficulties. For instance, prior research has shown reactive aggression to be associated with poor inhibitory control and planning [40]. In addition, because reactive aggression is associated with social cognitive deficits [52], it may be that these children have difficulties in cognitive flexibility, which is the adaptive ability to adjust thinking and behavior in response to changing environmental conditions [53]. Further, compared to proactive aggression, reactive aggression has been associated with poorer problem solving [52], which may indicate deficits in concept formation [54]. In contrast, children who use both proactive and reactive aggression may display somewhat similar EF profiles of children who only engage in reactive aggression (i.e., poor inhibitory skills). However, they may have an intact cognitive ability to plan, problem-solve, and switch between operations—mental states that facilitate carrying out planned and predatory aggression. Yet, this possibility has yet to be examined.

### The Present Study

This is the first known study to include neurobiological and executive functioning measures to understand how subgroups of aggressive children differ. Based on prior research using person-centered analyses to identify subgroups, a latent class analysis was used to identify three hypothesized aggression groups based on self-report. We expected to find three classes in line with previous research, which finds a low aggressive group, a reactively aggressive group, and a mixed group [9]. It was expected that psychophysiological, neuropsychological, and personality factors would drive group differences. For psychopathic traits, high levels of CU traits were expected to increase the likelihood of being in the mixed group when compared to the reactive and low group. High levels of impulsive psychopathic traits were expected to increase the likelihood of being in the reactive group and mixed group over the low group. It was expected that low resting RSA would increase the likelihood of being in the mixed group, when compared to the low and reactive group. To investigate differences in executive function, four tests were selected from the Delis–Kaplan Executive Function System (D–KEFS) to differentiate the aggression groups; The Color Word Inference Test (inhibitory control), Tower Test (planning), Trail Making Test (Cognitive Flexibility), and Sorting Test (Concept Formation). We expected poor performance on all four EF measures to increase the likelihood of being in the reactive group, when compared to the low group. Because proactive aggression is thought to require more complex cognitive processes, we expected better performance on planning, cognitive flexibility, and concept formation to increase the likelihood of being in the mixed group when compared to the reactive group. Since the mixed group display reactive aggression, we also expected poor inhibitory control to increase the likelihood of being in the mixed group compared to the low group. Thus, across two measures, which broadly assess prefrontal functioning [1, 43], we expected the reactive aggression group to perform most poorly on EF when compared to the low aggression group. Yet, because of the research on CU traits and RSA, we expected the mixed group to be lowest on RSA but possibly similar to the low group on the Tower Test, Trail Making Test, and Sorting Test.

## Method

### Participants

Sixty boys and 50 girls (*N* = 110, *M*_age_ = 9.9 years, *SD* = 0.71, age range: 9–11 years) were recruited from two primary schools in the North East of England. Typically, aggression research has focused on low socioeconomic groups because of the link between low SES and aggressive behavior [55]. However, our aim was to assess aggression in a normative sample. Free school meals are considered a reliable estimate of socioeconomic status [56], therefore, to ensure our sample was normative, we selected schools within the national average (in the third quintile) of receiving free school meals. Participant ethnicity was reported by the child’s teacher, with the sample including White British (96%), Black British (2%), and Asian British (2%). Using the Wechsler Abbreviated Scale of Intelligence (WASI-II) the mean full scale IQ (FSIQ-2) for the sample was 98.28 (*SD* = 11.65). Children who would be between the ages of 9–11 years at the time of study administration were included in the recruitment process. The recruitment success rate was 97%, with four students declining or unable to participate. Participants were excluded from the study if they were taking stimulant medication (methylphenidate; *n* = 1) or had visual impairments (*n* = 2) that would affect performance on executive functioning tasks.

### Procedure

Information sheets detailing the scope of the study and consent forms were sent home to caregivers 4 weeks prior to the beginning of the study. Reminders were sent home to parents of children who expressed interest but a consent form had not been received. Once parent consent was received assent was then obtained from the child. Participants were tested individually in a quiet room within the school. Psychophysiological assessment, self-report questionnaires, and IQ testing were administered during the same session. Executive functioning assessment occurred on a different day, but within 3 days of the first administration session. Participants received a small gift for participating in the study. The study was approved by the Ethics Committee at the University of Durham.

### Measures

#### Psychopathic Traits

The Antisocial Process Screening Device (APSD [20]) was completed by the child’s teacher to measure psychopathic traits. The APSD was designed to measure psychopathic traits in children and adolescents, and for use in both forensic and community populations [57]. The APSD consists of 20-items yielding a total score with each item rated on from 0 (Not at all true) to 2 (Definitely true). The APSD captures the dimensional construct of psychopathy, with five items measuring impulsivity (e.g., “Engages in risky or dangerous activities”), seven items for narcissism (e.g., “Uses or cons other people to get what s/he wants”), and six items representing callous–unemotional traits (e.g., “Does not show feelings or emotions”). The Cronbach’s alpha coefficient for APSD total score (0.91), and the three dimensions (narcissism = 0.87; impulsivity = 0.80; callous–unemotional = 0.84) suggests good reliability.

#### Self-Report of Reactive and Proactive Aggression

Participants completed the reactive–proactive aggression questionnaire (RPQ [19]). The RPQ is suitable for children with a reading age of 8 years. The 23-item scale captures physical and verbal reactive and proactive aggression. The reactive and proactive aggression subscales consists of 11 items (e.g., “Gotten angry or mad or hit others when teased”) and 12 items (e.g., “Hurt others to win a game”), respectively. Each item is reported on a three-point scale ranging from 0 (Never) to 2 (Often). The Cronbach’s alpha coefficient for the total (α = 0.89), reactive (α = 0.86), and proactive (α = 0.80) scales were considered good. The RPQ was used to identify groups of aggressors using a latent class analysis.

#### Teacher-Report of Reactive and Proactive Aggression

Teachers completed the Proactive/Reactive Aggression Scale (PRA [7]). The full scale consists of 6-items, with responses ranging from 1 (Never true) to 5 (Almost always true). The proactive scale (e.g., “This child uses physical force [or threatens to use force] in order to dominate other kids”) and reactive scale (e.g., “When this child has been teased or threatened, he or she gets angry and easily strikes back”) each consist of 3-items. The total score (α = 0.92), reactive (α = 0.94), and proactive (α = 0.93) subscales had good internal consistency. As expected, the RPQ and PRA reactive (0.54) and proactive (0.66) scales were moderate to highly correlated.

#### Intelligence Quotient

The Wechsler Abbreviated Scale of Intelligence second edition (WASI-II [58]) is a brief measure of intellectual functioning for people of ages 6–89 years. The present study used the FSIQ-2, which is an estimate of intelligence comprised of the Vocabulary (a measure of comprehension knowledge) and Matrix Reasoning (a measure of fluid reasoning) subtests. In youth, the FSIQ-2 [58] has been found to have good internal consistency (0.93) and test–retest reliability (0.85). Correlations among the FSIQ-2 and the Wechsler Intelligence Scale for Children-III (WISC-III) have been high (0.85). Administration time was approximately 15 min for each child.

### Executive Function

The D–KEFS [54] was administered as a test battery for measures of executive functioning; planning, rule learning, concept formation, inhibition, and cognitive flexibility. The D–KEFS is a widely used clinical neuropsychological and research measure of executive functioning, and is age appropriate for children as young as 8 years. To account for participant age, scaled scores were used for all measures.

#### Tower Test

The D–KEFS Tower Test [54] measures planning, goal setting, rule learning, problem-solving, and perseverative responding skills. Using different colored and sized wooden disks, participants were asked to stack the disks on one of three wooden pegs to match a picture. Participants were instructed to follow two rules, they were to move only one disk at a time, and a large disk could not be placed on a smaller disk. Further, the participant was informed that the aim of the task was to create the picture (which remained opposite the participant throughout the condition) in as fewest amount of moves possible. Before the participant began the condition, disks were placed in a prearranged order (starting position) and a new picture was presented (ending position; see examiners manual for more details [54]). There were a total of nine conditions, with each condition increasing in difficulty. Performance on the Tower Test was calculated based on a Total Achievement scaled score, which takes into account the number of moves used to complete the condition. Higher scores indicate better performance. Administration time was approximately 15 min per child.

#### Color Word Inference Test

The D–KEFS color word inference test (CWIT) measures inhibitory control [54]. The CWIT consists of three testing conditions, each condition was timed and the participant was asked to complete the condition as quickly as they could without making any errors. In the first condition the participant was asked to verbally name a series of color squares, and in the second condition read aloud a series of words written in black ink, these words were the name of colors (i.e., blue, green). Condition one and two serve as a baseline, and are used to calculate difference scores for condition three. The third condition is similar to the classic stroop procedure that measures inhibition [54]. The participant named the color of the ink the word was printed in (e.g., “blue” for a word written in blue colored ink). However the ink color of the word was incongruent to the written word (i.e., the word blue was written in red ink). In accordance to the user manual [54], completion time difference scaled scores were used. Higher scores indicate better performance. The CWIT took approximately 10 min to administer for each child.

#### Trail Making Test

The D–KEFS Trail Making Task (TMT [54]) assessed cognitive flexibility, an ability to switch back and forth between tasks, operations, or mental states. The TMT consists of five different conditions; visual scanning, number sequencing, letter sequencing, number-letter switching, and motor speed. Conditions were presented on a 11 × 17 inch area. The first three tests (visual scanning, number sequencing, and letter sequencing) measure fundamental abilities that are needed to complete the number-letter switching. The visual scanning condition requires the participant to cross out all the 3s on the paper, which are amongst other numbers and letters. The number sequencing condition requires the participant to draw a line from circles numbered from 1 to 16 in numerical order. The letter sequencing condition requires the participant to draw a line from circles lettered from A–P in alphabetical order. The number–letter switching conditions requires the participant to connect numbers and letters in an alternating switching order (i.e., 1-A-2-B-3-C-4…) until the participant reaches the final letter (P). The last condition, motor speed, requires the participant to trace over a dotted line that connects empty circles. The aim for all conditions is to complete the task as quickly as possible without making any mistakes. Completion time difference scaled scores were used; higher scores indicate better performance.

#### Sorting Test

The D–KEFS Sorting Test (DST [54]) is a measure of concept formation and problem-solving. Participants sorted six cards into two groups of three cards. The card set had eight possible combinations of sorts (e.g., size, color, shape, meaning of words printed on the cards). The number of correct sorts were summed for a total score (number of correct sorts). Higher scores indicate better performance. The DST was designed to reduce the role on inhibitory control so that concept formation is the focus measure [59].

### Psychophysiological Recording and Reduction

Respiration and electrocardiogram (ECG) were recorded continuously over a 2-min rest period at 1000 Hz using Biopac system (MP150-BIOPAC Systems Inc., Goleta, CA) connected to a MacBook Pro running AcqKnowledge software version 4.3 (Biopac Systems). Respiration and ECG were recorded using BioNomadix module transmitter, which was secured around the child’s chest. Recorded data were analyzed offline using AcqKnowledge 4.3 software (BIOPAC Inc.). Respiration was recorded using RSPEC-R amplifier with a wireless respiration belt transducer. The belt was placed at maximum point of sensitivity - the child was asked to exhale, at full exhalation the respiration belt was fastened around the child’s abdomen. To measure cardiac activity, participants were fitted with three self-adhesive pre-jelled Ag–AgCL ECG electrodes using the standard lead-II configuration (distal right collarbone, lower left rib, and lower right rib [ground]). During a 10 min stabilization period, participants completed questionnaires. Participants were asked to relax and sit still for two min.

#### Respiratory Sinus Arrhythmia

RSA was calculated to measure resting parasympathetic activity. ECG data were resampled at 250 Hz. ECG waveforms were visually inspected for artifacts. RSA was derived from the respiration amplifier (RSPEC-R) with a band-pass frequency fixed at 0.707 and 0.05 Hz. RSA was computed using AcqKnowledge automated function for RSA analysis, which applies the validated peak–valley method [60]. RSA values reflect the millisecond difference between the minimum and maximum R–R intervals during each respiration cycle. Lower vagal activity is reflected by lower RSA values, and higher vagal activity is reflected by higher RSA values [61].

### Data Analytic Plan

To identify groups of aggressors a latent class analysis (LCA) was conducted with MPlus 7.2 [62], using the reactive and proactive aggression subscales of the Reactive–Proactive Questionnaire (RPQ). LCA is a person-centered latent variable method that, within a heterogeneous sample, is able to identify groups of individuals. Multiple fit indices were used to identify the best fitting model. Fit indices included the Lo–Mendel–Rubin Adjusted Likelihood Ratio Test (LMRT), Akaike Information Criteria (AIC), Bayesian Information Criteria (BIC), sample-size adjusted BIC (ABIC), entropy values, and mean posterior probabilities. LMRT assesses if the model with k classes provides a significantly (*p* < 0.05) better fit than the model with k–1 classes. If the LMRT did not reach significance (*p* > 0.05), the model with k–1 fewer latent classes was selected. Additionally, the model with the lowest AIC and BIC suggests a better fitting model. Values for entropy and posterior probabilities of latent class membership range from 0 to 1, with higher values indicating more accurate classification of individuals. Average posterior classification probabilities should exceed 0.70 [63]. The RPQ (self-report) was used over the PRA (teacher-report) for several reasons. Later analyses included teacher-report of psychopathic traits, therefore using child-report of aggression to classify groups would provide a multi-informant representation of the individual; a multi-informant approach is considered a more accurate reflection of the child’s behavior [64]. Further, the RPQ has many more items than the PRA (23-items, 6-items, respectively), allowing respondents to have greater variance in their answers increasing the likelihood of finding a less restricted reflection of aggression groups. Further, the RPQ has been widely validated cross-culturally and used extensively in psychophysiological research [65]. Univariate analysis of variance (ANOVA) were conducted to test if the aggression groups accurately reflected the teacher ratings of proactive and reactive aggression. To examine the likelihood of a child belonging to an aggression group based on levels of psychopathic traits, RSA, and performance on EF measures, three multinomial logistic regressions (MLR) were conducted. When testing psychopathic traits and RSA, the models included age and sex as covariates. When testing EF measures, IQ and sex were controlled for. Age was not included as a covariate because the scaled scores account for age [54].

## Results

### Latent Class Analysis

Table 1 presents the fit indices for the 2-through 4-latent class models for reactive and proactive aggression groups. The AIC, BIC, ABIC, and LMRT favored the 3-class model over the 2-class model. When comparing the 4-to the 3-class model the AIC, BIC, and ABIC favored the 4-class model; however, these differences were marginal. When comparing 4- to the 3-class model on the entropy value, the 3-class model was preferred. The LMRT indicates the 3-class model was a significantly better fit than the 2-class model, but the 4-class model was not a significantly better fit than the 3-class model. This suggests the best model for the data was the 3-class. Further, the 3-class model classified people with a high degree of accuracy (low aggression group 0.97; reactive aggression group 0.88; and mixed aggression 0.99). Importantly, the 3-class model was most similar to prior research, which has also found the 3-class model [8]. Further, the proportion of participants in the mixed (*n* = 10, 9%), reactive (*n* = 28, 25.5%), and low aggression group (*n* = 72, 65.5%) was consistent with prior studies that include community samples of adolescents [16], and a slightly lower proportion than detained samples [9].

**Table 1:** Fit indices for latent class models of self-report proactive and reactive aggression

Classes	AIC	BIC	ABIC	Entropy	LMRT
2	1155.59	1174.49	1152.37	0.92	75.95
3	1120.93	1147.93	1116.33	0.89	37.97***
4	1107.67	1142.77	1101.69	0.88	17.99

*AIC* Akaike information criteria, *BIC* Bayesian information criteria, *ABIC* sample-size adjusted Bayesian information criteria, *LMRT* Lo–Mendel–Rubin adjusted likelihood ratio test

***p < 0.001

The low aggression group was lower on self-report of reactive (*M* = 7.11, *SD* = 3.63) and proactive aggression (*M* = 0.81, *SD* = 0.91) than the reactive (*p* < 0.001, *p* < 0.001, respectively) and the mixed group (*p* < 0.001, *p* < 0.001, respectively). The mixed group scored higher than the reactive group on reactive (*M* = 16.40, *SD* = 3.69; *M* = 11.25, *SD* = 3.46, *p* = 0.001, respectively) and proactive aggression (*M* = 11.00, *SD* = 1.69; *M* = 4.75, *SD* = 1.38, *p* < 0.004, respectively). Aggression groups differed significantly on teacher-report of reactive (*F* (2, 107) = 15.92, *p* < 0.001, η_p_^2^ = 0.23) and proactive aggression (*F* (2, 107) = 30.59, *p* < .001, η_p_^2^ = 0.37). Post hoc Games-Howell comparisons indicated the reactive group (*M* = 5.57, *SD* = 2.87) was significantly higher than the low group on reactive aggression (*M* = 3.99, *SD* = 1.53, *p* = 0.024). Further, the reactive group (*M* = 4.79, *SD* = 2.89) was higher than the low group on proactive aggression (*M* = 3.39, *SD* = 0.99, *p* = 0.047). The mixed group was significantly higher in reactive (*M* = 7.50, *SD* = 2.76, *p* = 0.007) and proactive aggression (*M* = 8.60, *SD* = 3.98, *p* = 0.006) than the low aggression group. Compared to the reactive group, the mixed was significantly higher on proactive aggression (*p* = 0.04) but not reactive aggression (*p* = 0.177).

### Psychopathic Traits

We conducted a multinomial logistic regression (MLR) to test the hypotheses that psychopathic traits would differentiate the aggression groups. Specifically, high CU traits would differentiate the mixed group from the low and reactive groups, while impulsive psychopathic traits would differentiate the reactive and mixed group from the low group. Table 2 includes the results from the MLR, with odds ratios and confidence intervals. Odds ratios reflect the odds likelihood of being in one group over the other, based on the level of the independent variable. The MLR comparing psychopathic traits was significant, χ^2^ (10, *N* = 110) = 51.40, *p* < 0.001. Sex and age did not increase the likelihood of being in one group over another. Children who scored high on impulsivity were more likely to be the reactive group and mixed group when compared to the low group. However, impulsivity did not increase the likelihood of being in the mixed group when compared to the reactive group. Children scoring high on CU traits were more likely to be in the mixed group, when compared to the reactive and low group. Narcissistic psychopathic traits did not significantly increase the likelihood of being in one group over another.

**Table 2:** Aggression group comparisons on psychopathic traits based on odds ratios (95% CI)

	Low group versus reactive group^a^	Low group versus mixed group^a^	Reactive group versus mixed group^b^
Sex	0.55 (0.19, 1.61)	0.08 (0.00, 1.74)	0.15 (0.01, 3.09)
Age	0.88 (0.45, 1.71)	1.70 (0.36, 8.17)	1.95 (0.41, 9.20)
Callous–unemotional	1.06 (0.81, 1.39)	2.51** (1.30, 4.85)	2.36** (1.24, 4.52)
Narcissism	0.80 (0.61, 1.04)	0.59 (0.34, 1.01)	0.74 (0.43, 1.27)
Impulsivity	1.58** (1.16, 2.16)	2.37** (1.31, 4.28)	1.50 (0.88, 2.57)

*CI* confidence interval

**p < 0.01

^a^Reference category is low aggression group

^b^Reference category is reactive aggression group

### Psychophysiological Profiles

To test the hypothesis that low resting RSA would differentiate the mixed group from the low and reactive groups we conducted a MLR. Table 3 includes the results from the MLR, which included sex, age, and RSA. The MLR was significant, χ^2^(6, *N* = 110) = 16.98, *p* = 0.009. Age and sex did not significantly increase the odds of being in one group over another. Consistent with our hypothesis, children with low resting RSA were more likely to be in the mixed group when compared to both the reactive and low group. Thus, RSA did not differentiate the reactive group from the low group.

**Table 3:** Aggression group comparisons on respiratory sinus arrhythmia based on odds ratios (95% CI)

	Low group versus reactive group^a^	Low group versus mixed group^a^	Reactive group versus mixed group^b^
Sex	1.05 (0.42, 2.59)	2.02 (0.45, 9.00)	1.93 (0.37, 10.00)
Age	0.77 (0.41, 1.44)	1.74 (0.56, 5.37)	2.63 (0.67, 7.70)
RSA	1.55 (0.78, 3.05)	0.16** (0.05, 0.51)	0.10*** (0.03, 0.37)

*CI* confidence interval

**p < 0.01; ***p = 0.001

^a^Reference category is low aggression group

^b^Reference category is reactive aggression group

### Executive Functioning Profiles of Aggression Groups

Table 4 displays the means and standard deviations of the EF functioning measures by aggression group. A final MLR was conducted to test the hypotheses that the reactive group would be differentiated from the low group by poor performance on inhibitory control (color word inference test), planning (tower test), cognitive flexibility (trail making test), and concept formation (sorting test) as measured by the D–KEFS. Further, we expected poor performance on inhibitory control to increase the likelihood of being in the mixed group when compared to the low group. When comparing the two aggression groups, we expected better performance on planning, concept formation, and cognitive flexibility to increase the likelihood of being in the mixed group, when compared to the reactive group.

**Table 4:** Means and standard deviation of executive functioning tests by group

	Low Group	Reactive Group	Mixed Group
IQ	99.24 (11.49)	99.64 (12.66)	96.00 (11.65)
Planning	11.22 (1.73)	9.61 (2.17)	11.80 (2.49)
Inhibitory control	11.35 (2.39)	9.43 (2.03)	11.70 (1.77)
Concept formation	9.28 (2.56)	7.68 (2.99)	8.30 (3.27)
Cognitive flexibility	10.69 (3.527)	9.79 (2.33)	10.90 (3.32)

*Planning* tower test, *Inhibitory control* color word inference test, *Concept formation* sorting test, *Cognitive flexibility* trail making test

Table 5 includes the results from the MLR, which included sex, IQ, planning, inhibition, concept formation, and cognitive flexibility. The model was significant, χ^2^ (12, *N* = 110) = 32.46, *p* = 0.001. Sex, IQ, concept formation, and cognitive flexibility did not increase the likelihood of a child being in one group over another. However, children scoring worse on planning and inhibition were more likely to be in the reactive group when compared to the mixed and low aggression groups. None of the EF measures increased the likelihood of being in the low group compared to the mixed group. Thus, the reactive group showed poor performance on areas of planning and inhibitory control, yet the mixed group was similar to the low aggression group.

**Table 5:** Aggression Group Comparisons on Executive Function Based on Odds Ratios (95% CI)

	Low group versus reactive group^a^	Low group versus mixed group^a^	Reactive group versus mixed group^b^
Sex	1.32 (0.47, 3.68)	1.31 (0.33, 5.27)	0.99 (0.20, 4.95)
IQ	1.02 (0.97, 1.07)	0.99 (0.92, 1.05)	0.97 (0.90, 1.04)
Planning	0.69* (0.51, 0.94)	1.17 (0.82, 1.67)	1.70* (1.09, 2.66)
Inhibitory Control	0.67** (0.51, 0.87)	1.06 (0.80, 1.42)	1.60* (1.09, 2.33)
Concept Formation	0.87 (0.70, 1.07)	0.87 (0.67, 1.14)	1.01 (0.74, 1.38)
Cognitive Flexibility	0.93 (0.78, 1.11)	0.99 (0.79, 1.23)	1.06 (0.81, 1.37)

*Planning* tower test, *Inhibitory control* color word inference test, *Concept formation* sorting test, *Cognitive flexibility* trail making test, *CI* confidence interval

*p < 0.05; **p < 0.01

^a^Reference category is low aggression group

^b^Reference category is reactive aggression group

## Discussion

Prior research suggests psychopathic traits, neurobiology, and executive functioning play an important role in defining subtypes of aggressive behavior [40], and the present findings suggest this is also true for aggressive subgroups of typically developing preadolescent children. Specifically, children who displayed executive function deficits were more likely to be in the reactive aggression group, whereas children who scored high on CU traits and had lower resting RSA were more likely to be in the mixed aggression group. This suggests children who are only aggressive in response to provocation may have attention modulation difficulties, as evidenced by executive function difficulties. In contrast, children who show mixed forms of aggression demonstrate intact neurocognitive skills, skills that facilitate the successful implementation of top–down goal-directed behavior [66]. However, these same children may have neurobiological differences (as indexed by low resting RSA) that are characteristic of youth high on psychopathic traits [67].

Deficits in EF have a fairly robust association with aggression [4, 40, 42], however, based on theory [13] and the present study, this may be limited to children who only reactively aggress. In particular, poor performance on inhibitory control and planning increased the likelihood of children being in the reactive group when compared to the low and mixed aggression groups. It is important to note, neither concept formation nor cognitive flexibility differentiated the reactive group from the mixed or low group. These null findings are consistent with variable-centered research in children and adults [40, 68], and implies the ability to shift attention between tasks or mental states and solve problems are not core deficits found in reactively aggressive children. The present findings demonstrate two important implications: (i) children who only engage in reactive aggression are cognitively different from children who engage in mixed and low aggression, and (ii) deficits in inhibitory control and planning may be specific to children who only reactively aggress [40].

Although the mixed group displayed similarly high levels of reactive aggression compared to the reactive group, children in the mixed group can be characterized by intact EF skills. This is not surprising because the neuroscience of proactive aggression has been suggested to be more complex than that of reactive aggression [13]. Thus, intact EF ability may explain why some children are able to implement drawn-out goal-directed aggression. Therefore, the reactive aggression displayed by the mixed group is not a result of poor EF ability. Instead, a possible explanation may be due to impulsive psychopathic traits. Children with high levels of impulsive psychopathic traits were more likely to be in the mixed and reactive groups compared to the low group. Thus, impulsive psychopathic traits (e.g., risk-taking and dangerous behavior, blaming others, and easily bored) may explain the overlap between children in the reactive and mixed aggression groups. This indicates there are similarities between groups at the personality/behavioral level.

Using a person-centered model, low RSA, high CU traits, and intact EF increased the likelihood of children exhibiting high levels of both proactive and reactive aggression. Children in the mixed aggression group may be able to proactively aggress because of their neuropsychological, neurobiological, and personality profiles. These children display low levels of RSA, an efferent marker of PFC function [1]. RSA is suggested to have an integral role in social adjustment [69]; children and adolescents with low RSA have high levels of behavioral problems and psychopathic traits [39, 70]. Therefore, resting RSA may serve as neurobiological marker to differentiate groups of highly aggressive children. Further, children in the mixed group displayed higher levels of CU traits, which may explain their emotional detachment and willingness to hurt others for their personal gain. When making moral decisions, youth with psychopathic traits have reduced functional connectivity between the amygdala and orbitofrontal cortex [71]. Hypoactive connectivity may implicate the development of reinforcement learning associated with moral decision-making, which could explain why children with CU traits positively appraise outcomes from aggression [72, 73], promoting the use of proactive aggression. In support of this link, reduced amygdala activity to emotional distress cues have been found to mediate the association between CU traits and proactive aggression [74]. The present findings support this earlier research, whereby children in the mixed aggression group, who are higher on CU traits, are neurobiologically distinct from children who only reactively aggress. These children also have the cognitive ability to successfully implement top–down goal-directed behavior, in this case proactive aggression. These differences may explain how a small number of children can carry out premeditated, instrumental, and “cold-blooded” aggression [7, 75, 76].

The present findings must be interpreted with several limitations in mind. While the sample and group sizes were consistent to prior research using similar indices [8], and the focus of the study was typically developing children, there may be a lack of generalizability to children with more serious levels of aggressive behavior. Thus, future research using the similar multidisciplinary indices in clinical samples may be warranted to explore the replicability of the findings in children with serious behavioral problems. Further, in this preadolescent sample pubertal timing was not accounted for, which has been shown to be associated with RSA [33] and externalizing behaviors [77]. Even with these limitations in mind, meaningful results have been found. This is the first known study to begin to elaborate on the co-occurrence of proactive and reactive aggression in preadolescents, and to demonstrate how these children differ across cognitive, neurobiological, and psychopathic indices from children who display only reactive forms of aggression. Also, using a multi-informant approach, the three aggression groups found in prior adolescent samples were confirmed with preadolescent children.

Aggressive behavior in young children is typical but as children develop they start to better regulate emotions and selectively inhibit aggressive behaviors [3]. However, there are small numbers of children who continue to display high levels of aggression [5]. Early identification of aggressive groups of children and understanding the cognitive, neurobiological, and psychological profiles is an important endeavor for advancing and individualizing early intervention. The present findings suggest that aggressive children are indeed a heterogeneous group, and therefore early interventions may be best tailored to the child’s needs based on the aggression displayed. For instance, young children who display only reactive aggression may benefit from interventions targeting impulsivity, inhibitory control and planning ability. In contrast, children who use both proactive and reactive forms of aggression may not benefit from the same interventions, as they do not display the same cognitive deficits. Instead, these children are characterized by low RSA and high CU traits. Therefore, the needs of these children may be best addressed by interventions shown to be effective for children with CU traits–interventions which reward friendship, emotion regulation, and perspective taking [78].

### Summary

A person-centered approach to reactive and proactive aggression has yet to be conducted in children. Identifying groups of children based on their use of reactive and proactive aggression is essential for understanding individuals, and informing intervention and treatment programs. Examining group differences on neurobiological functioning, executive functioning, and personality may help explain the mechanisms by which children come to be reactive and proactive aggressors. A latent class analysis confirmed three groups of aggressors - a low aggression group, a high reactive aggression group, and a mixed aggression group (high reactive and proactive). Thus, in a small group of children, proactive and reactive aggression were found to co-occur. This finding supports and extends prior person-centered aggression research in adolescents. Importantly, meaningful differences were found between the groups. Children in the mixed aggression group were distinct from reactively aggressive children, by their high levels of callous–unemotional traits, low resting respiratory sinus arrhythmia, and intact executive functioning. Whereas children in the reactive group were distinct from children in the mixed aggression and low aggression groups by specific executive functioning deficits—planning and inhibitory control. These findings highlight psychobiological and executive functioning differences that may explain heterogeneity in childhood aggression.
